# Attack of the clones: whole genome-based characterization of two closely related enterohemorrhagic *Escherichia coli* O26 epidemic lineages

**DOI:** 10.1186/s12864-018-5045-7

**Published:** 2018-08-31

**Authors:** Lucia Karnisova, Monika Marejkova, Hana Hrbackova, Alexander Mellmann, Helge Karch, Angelika Fruth, Pavel Drevinek, Kveta Blahova, Martina Bielaszewska, Jaroslav Nunvar

**Affiliations:** 1 Department of Paediatrics, 2nd Faculty of Medicine, Charles University and Motol University Hospital, Prague, Czech Republic; 2 National Reference Laboratory for E. coli and Shigella, National Institute of Public Health, Prague, Czech Republic; 3 Laboratory for Tissue Cultures, National Institute of Public Health, Prague, Czech Republic; 4 Institute for Hygiene and the National Consulting Laboratory on Hemolytic Uremic Syndrome, University of Münster, Münster, Germany; 5 National Reference Center for Salmonella and Other Enteric Pathogens, Robert Koch Institute, Wernigerode, Germany; 6 Department of Medical Microbiology, 2nd Faculty of Medicine, Charles University and Motol University Hospital, Prague, Czech Republic

**Keywords:** Shiga toxin, O26, Enterohemorrhagic *Escherichia coli* (EHEC), New European clone

## Abstract

**Background:**

Enterohemorrhagic *Escherichia coli* (EHEC) O26:H11/H^−^, the most common non-O157 serotype causing hemolytic uremic syndrome worldwide, are evolutionarily highly dynamic with new pathogenic clones emerging rapidly. Here, we investigated the population structure of EHEC O26 isolated from patients in several European countries using whole genome sequencing, with emphasis on a detailed analysis of strains of the highly virulent new European clone (nEC) which has spread since 1990s.

**Results:**

Genome-wide single nucleotide polymorphism (SNP)-based analysis of 32 EHEC O26 isolated in the Czech Republic, Germany, Austria and Italy demonstrated a split of the nEC (ST29C2 clonal group) into two distinct lineages, which we termed, based on their temporal emergence, as “early” nEC and “late” nEC. The evolutionary divergence of the early nEC and late nEC is marked by the presence of 59 and 70 lineage-specific SNPs (synapomorphic mutations) in the genomes of the respective lineages. In silico analyses of publicly available *E. coli* O26 genomic sequences identified the late nEC lineage worldwide. Using a PCR designed to target the late nEC synapomorphic mutation in the *sen*/*ent* gene, we identified the early nEC decline accompanied by the late nEC rise in Germany and the Czech Republic since 2004 and 2013, respectively. Most of the late nEC strains harbor one of two major types of Shiga toxin 2a (Stx2a)-encoding prophages. The type I *stx*_2a_-phage is virtually identical to *stx*_2a_-phage of EHEC O104:H4 outbreak strain, whereas the type II *stx*_2a_-phage is a hybrid of EHEC O104:H4 and EHEC O157:H7 *stx*_2a_-phages and carries a novel mutation in Stx2a. Strains harboring these two phage types do not differ by the amounts and biological activities of Stx2a produced.

**Conclusions:**

Using SNP-level analyses, we provide the evidence of the evolutionary split of EHEC O26:H11/H^−^ nEC into two distinct lineages, and a recent replacement of the early nEC by the late nEC in Germany and the Czech Republic. PCR targeting the late nEC synapomorphic mutation in *ent*/*sen* enables the discrimination of early nEC strains and late nEC strains in clinical and environmental samples, thereby facilitating further investigations of their geographic distribution, prevalence, clinical significance and epidemiology.

**Electronic supplementary material:**

The online version of this article (10.1186/s12864-018-5045-7) contains supplementary material, which is available to authorized users.

## Background

Enterohemorrhagic *Escherichia coli* (EHEC) O26:H11/H^−^ is the most common non-O157 EHEC serotype causing diarrhea and its systemic complication, the hemolytic uremic syndrome (HUS) worldwide [1–16]. In Europe, the proportion of EHEC O26 among all EHEC patients´ isolates has been increasing during past years [1] and in some countries, EHEC O26 is a more frequent cause of HUS than EHEC O157:H7 [12, 16]. HUS caused by EHEC O26 can be as severe as that caused by EHEC O157:H7 [13, 17–20] and there is no significant difference in the long-term outcome of HUS caused by EHEC O26 and EHEC O157 [18]. In addition to sporadic cases, multiple outbreaks caused by EHEC O26 have been reported throughout the world [15, 21–30].

The majority of EHEC O26 clinical isolates carry genes encoding Shiga toxins (Stx) of two major types including Stx1a and/or Stx2a [9, 26, 27, 31–33]. However, a subset of strains contain genes encoding Stx subtypes such as Stx2d [34, 35], Stx2b [15], or Stx2f [36]. The presence of *stx*_2a_ and/or production of Stx2a alone is a predictor for a severe disease including progression of the infection to HUS [31]. The *stx*_2a_-harboring EHEC O26 belong to two major multilocus sequence types (STs) ST21 or ST29, whereas strains harboring *stx*_1a_ alone or together with *stx*_2a_ belong to ST21 [9, 31]. The ST29 clade is considerably heterogeneous and consists of several clonal lineages which differ by *stx*_2_ alleles and plasmid gene profiles [9, 31, 37, 38]. A new highly virulent ST29 clone harboring *stx*_2a_ only was first reported in Germany in the mid-1990s [33] and since then it has rapidly spread throughout Europe [31]. Strains of this lineage termed a “new German clone” or a “new European clone” [31, 38] exhibit a particular plasmid virulence gene profile characterized by the presence of EHEC-*hlyA* (encoding EHEC hemolysin) and *etpD* (encoding a type II effector), while *katP* (encoding catalase-peroxidase) and *espP* (encoding serine protease EspP) genes are absent [31, 33]. In 2015, a distinct ST29 clonal lineage harboring *stx*_2a_ or *stx*_2d_ and lacking the plasmid-borne virulence genes (EHEC-*hlyA*, *katP*, *espP, etpD*) was reported in France as a “new French clone” [35, 38]. Recently, yet another ST29 clone, which harbors *stx*_2a_ only and differs from both the European and French clone by plasmid virulence gene profile (EHEC-*hlyA*+, *katP*-, *espP*+, *etpD*-*)* emerged in Japan [9]. This Japanese clone (designated ST29C1 clade) is phylogenetically unrelated to the new European clone (ST29C2 clade) and the French clone (ST29C3 clade) [9].

Although EHEC O26 are the most common cause of pediatric HUS in the Czech Republic [12], the clonal structure of these strains has not been investigated in detail. The aim of this study was to analyze the phylogenetic relationships and clonal structure of EHEC O26 isolated from patients in the Czech Republic by using whole genome sequencing (WGS) with a particular focus on ST29 strains. To put these data into a global context, the genomic sequences of the Czech isolates were compared with those of EHEC O26 isolated in other European countries and with *E. coli* O26 genomes present in GenBank.

## Results

### Whole genome-based clonal structure of EHEC O26 population

Initially, we performed WGS of 16 EHEC O26 strains isolated from patients with HUS or diarrhea without HUS in the Czech Republic during the period of 2006–2016. The STs and plasmid gene profiles were determined in silico from draft genome sequences. This revealed the presence of both major O26 phylogenetic lineages, ST21 (6 isolates) and ST29 (10 isolates), among Czech isolates (Additional file 1: Table S1). Five of the six ST21 strains harbored *stx*_2a_ only, and one contained *stx*_1a_ only; all of them displayed a plasmid-borne virulence gene profile (EHEC-*hlyA*+, *katP+*, *espP+*, *etpD*-*)* previously identified in ST21 strains [31] (Additional file 1: Table S1). Nine of the ten ST29 EHEC O26 Czech strains corresponded to the new European clone (nEC) as defined in [31], i.e., they contained *stx*_2a_ as the sole *stx* gene and the plasmid virulence gene profile EHEC-*hlyA*+, *katP*-, *espP-*, and *etpD*+ (Additional file 1: Table S1). Remarkably, the remaining ST29 strain (15–496) harbored the plasmid gene profile typical for the nEC, but combined with the presence of *stx*_1c_ gene only instead of *stx*_2a_ (Additional file 1: Table S1). This is, to the best of our knowledge, the first report of *stx*_1c_ genotype in strains of the nEC. To gain a more detailed insight into the population structure of the nEC, we performed additional WGS of a collection of European nEC isolates including 16 strains originating from Germany (*n* = 11), Italy (*n* = 3), and Austria (*n* = 2). For comparison, genomic sequences of *E. coli* O26 available in the GenBank database were included in this analysis, making up the final set of total 159 *E. coli* O26 isolates whose genetic relationships were examined (Additional file 1: Table S1).

**Fig. 1:**
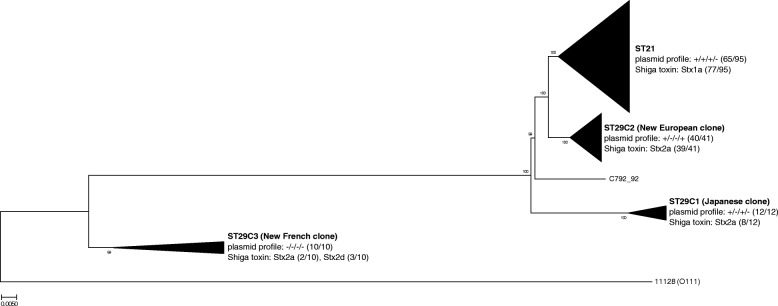
Global population structure of *E. coli* O26 based on whole genome phylogeny. The major lineages are named according to the current convention [9, 39]; strain C792_92 did not group within any lineage. The proportions of strains carrying plasmid gene profile characteristic for particular lineage (EHEC-*hlyA*/*katP*/*espP*/*etpD*) are denoted in parentheses. Predominant type of Shiga toxin and its proportion among strains of each particular lineage are denoted in parentheses. Core genome phylogeny was constructed from 12,205 variable bases among 3,718,610 validated homologous nucleotide positions. Phylogenetic tree was constructed with FastTree using approximately Maximum Likelihood phylogeny model [60]. Genomic sequence of *E. coli* O111:H^−^ strain 11128 (stripped of plasmids, prophages and other horizontally acquired regions [68]) was used as an outgroup. For additional information about particular strains, see Additional file 1: Table S1. For a complete (unreduced) phylogram, see Additional file 2: Figure S1

**Fig. 2:**
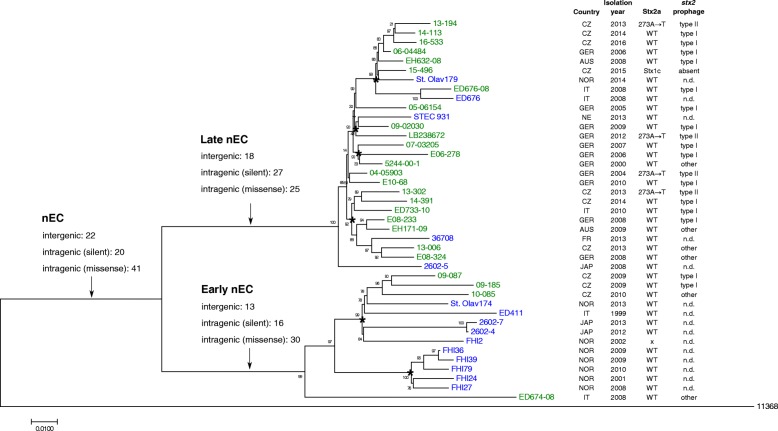
Detailed whole-genome phylogeny of *E. coli* O26 nEC (ST29C2). Numbers and categories of SNPs synapomorphic for early nEC, late nEC or nEC in general (Additional file 4: Table S2) are indicated above particular branches. Strains sequenced in this study are denoted in green, strains whose genomic sequences were obtained from other sources are denoted in blue. Nodes supported by both FastTree and Neighbor-Joining methods of tree inference are denoted with asterisks. Countries of origins are abbreviated as follows: AUS, Austria; CZ, Czech Republic; FR, France; GER, Germany; IT, Italy; JAP, Japan; NE, Netherlands; NOR, Norway. The type of *stx*_2a_-converting prophage present in particular strain is indicated as in Fig. 4; strains exhibiting significant deviations from coverage pattern and/or mutational pattern characteristic for type I or type II prophages are denoted as “other”. Types of *stx*_2a_-converting prophages were not determined for whole genome assemblies obtained from other sources (n.d.). Amino acid change in Stx2a (273A → T) specific for type II prophages is denoted. Core genome phylogeny was constructed from 1,258 variable bases among 4,332,095 validated homologous nucleotide positions. Phylogenetic tree was constructed with FastTree using approximately Maximum Likelihood phylogeny model [60]. Genomic sequence of *E. coli* O26:H11 strain 11368 (stripped of plasmids, prophages and other horizontally acquired regions [68]) was used as an outgroup

A genome-wide single nucleotide polymorphism (SNP)-based phylogram was constructed using the maximum likelihood model to provide high-resolution inference of *E. coli* O26 phylogeny (Fig. 1, Additional file 2: Figure S1). This phylogram clearly identified four major lineages, whose characteristics (i.e. ST, plasmid gene profile, and predominant Stx type) were in accordance with previous reports [9, 37–39]. Strikingly, the phylogenetic analysis demonstrated that strains of the nEC (syn. ST29C2 [9]) split into two distinct clusters (Fig. 2). This is in accordance with the observation of two distinct pulse-field gel electrophoresis (PFGE) clusters among a large, representative collection of European ST29 strains isolated between 1996 and 2012 in the original description of the nEC [31]. Retrospectively, we noted tentative differences between the two ST29 PFGE clusters with respect to the distribution of isolation dates of the corresponding strains. First strains of PFGE cluster B were isolated as early as in 1996 and their majority (53.6%) until 2004. In contrast, strains of the PFGE cluster C started to emerge, with a single exception, since 2004 [31]. We therefore propose to name the WGS lineages homologous to the PFGE clusters B and C as “early” and “late” nEC, respectively (Fig. 2, Additional file 3: Table S3). This designation is further supported by a detailed analysis of a temporal shift between the nEC lineages, as shown below.

### Evolution, diversification and spread of the nEC

To infer which genetic events underlied the evolutionary establishment of the nEC and its subsequent split into the “early” and “late” lineages, we sought mutations characteristic for particular lineages, i.e., SNPs absent in other *E. coli* O26 phylogroups including ST21 and ST29 non-nEC strains (synapomorphic mutations; for details, see Methods). The sets of synapomorphic mutations detected by this approach (Additional file 4: Table S2) provide an unambiguous genetic definition of the particular nEC lineages. The total numbers of SNPs synapomorphic for early nEC, late nEC and nEC as a whole were 59, 70, and 83, respectively (Fig. 2). This justifies that the early nEC and late nEC should be considered distinct phylogenetic entities. Next, we focused on parallel evolution of missense synapomorphic mutations (which are likely to influence function of encoded proteins) within the nEC. Three cases where a single gene underwent multiple amino acid changes during various phases of nEC evolution were detected: cation transporter CusA and hypothetical proteins YehI and YggM accumulated two mutations each. Four operons accumulated two missense mutations each: *glgBXCAP* (glycogen metabolism), *wza-wzb-wzc* (capsular polysaccharide synthesis), *hisJQMP* (histidine transport) and *gcl-hyi-glxR-ybbW-allB-ybbY-glxK* (purine metabolism) (Additional file 4: Table S2). Notably, three mutations in fimbrial biogenesis proteins (ElfG, YqiG, and HtrE) accounted for 12% of 25 missense mutations synapomorphic for the late nEC (Additional file 4: Table S2). Since fimbriae are determinants of bacterial adherence, their modification might affect persistence of late nEC strains in mammal hosts or even pathogenesis in infected humans.

**Fig. 3:**
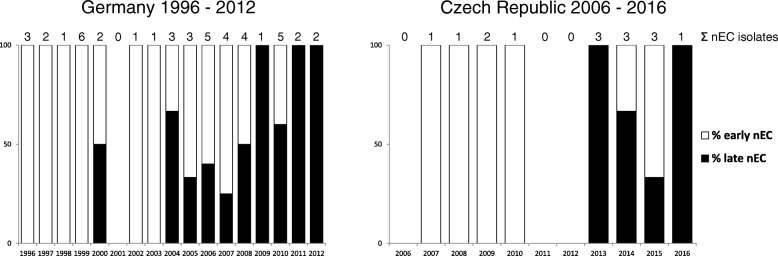
Annual incidence of early nEC and late nEC isolates in two Central European countries. The charts depict proportions of early nEC and late nEC among nEC isolates (*E. coli* O26, ST29, EHEC-*hlyA*+, *katP*-, *espP-*, *etpD*+) collected in Germany and Czech Republic during time periods indicated. Early nEC and late nEC isolates were discriminated by the *sen/ent* SNP-specific PCR (see Methods and Additional file 3: Table S3)

To rapidly differentiate between early nEC and late nEC strains, we designed a simple SNP-based PCR, which discriminates a mutation in the *sen*/*ent* gene (encoding *Shigella flexneri* enterotoxin 2 [40]), which is synapomorphic for the late nEC (Additional file 4: Table S2) (see Methods). This PCR was applied to screen all nEC isolates originating from Germany and the Czech Republic (45 and 15 isolates, respectively; Additional file 3: Table S3). Despite the limited numbers of isolates, a trend of the early nEC decline with a concomitant increase in the late nEC proportion was observed in both countries (Fig. 3). This suggests that the two lineages might occupy the same niche and compete with each other. The emergence of the late nEC in Germany (regularly isolated since 2004) preceded its first occurrence in the Czech Republic (2013) by nine years (Fig. 3). Strains belonging to the late nEC were also isolated in several other European countries including Austria (2008), Italy (2008, 2010), France (2013), Netherlands (2013) and Norway (2014), as well as in Japan (2008) (Fig. 2), which documents the pan-European and inter-continental distribution of this lineage.

### nEC strains harbor different *stx*_2a_-converting prophages

**Fig. 4:**
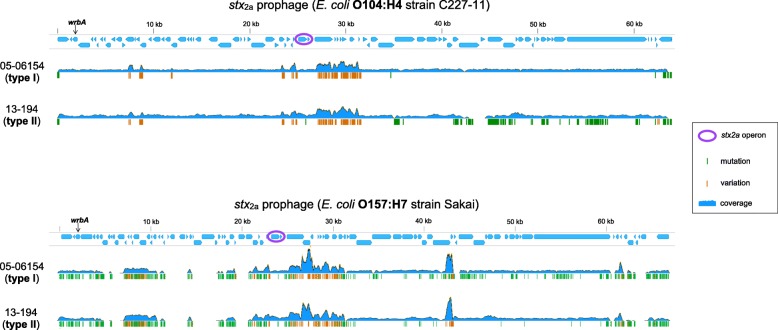
Different *stx*_2a_-converting prophages in EHEC O26 nEC strains. Visualization of sequencing reads mapped onto *stx*_2a_-converting prophages from *E. coli* O104:H4 strain C227–11 (GenBank accession: CP011331; *top*) and *E. coli* O157:H7 strain Sakai (GenBank accession: NC_002695; *bottom*). Strains 05–06154 and 13–194 represent the two distinct patterns of *stx*_2a_-converting prophages (type I and type II, respectively), which were detected among EHEC O26 nEC strains (see Fig. 2). Green vertical lines represent true mutations with respect to reference sequence. Orange vertical lines represent hybrid positions containing variant bases, which coincide with regions of abnormally high sequencing coverage (plausibly representing contributions of sequencing reads from other lambdoid prophages [68])

Shiga toxins encoded by *stx* genes are responsible for systemic complications of EHEC infections such as HUS. The *stx* genes are located in the genomes of lambdoid prophages (*stx-*converting phages) which are very heterogeneous (reviewed in [41]). A recent report [38] demonstrated that *stx*_2a_-converting prophages of EHEC O26 nEC strains were virtually identical with the *stx*_2a_-prophages present in the highly virulent *E. coli* O104:H4 German outbreak strain [42], while other O26 lineages harbored different *stx*-converting prophages [38]. To gain a deeper characterization of these elements in the early nEC and late nEC strains, we analyzed *stx*-converting prophages in all nEC genomes sequenced in this study, utilizing short sequencing reads and complete prophage sequences as references (Fig. 4). All *stx*_2a_-converting prophages in nEC strains were integrated into the *wrbA* gene which is also the site of phage integration in *E. coli* O104:H4 [43] and EHEC O157:H7 prototype strains Sakai [44] and EDL933 [45]. In agreement with Delannoy et al. [38], our results demonstrate that *stx*_2a_-converting prophages virtually identical with *E. coli* O104:H4 prophage (termed here “type I”) are predominant among nEC isolates. Unexpectedly, another distinct type of *stx*_2a_-converting prophage (termed here “type II”) was identified in four late nEC strains (Fig. 2). While the left half (5´-portion) of the type II prophage sequence was identical with O104:H4 prophage, the right half (3´-portion) diverged significantly (Fig. 4). The consensus sequences obtained by mapping of sequencing reads to the O104:H4 prophage reference were extracted from the divergent regions and searched against the BLAST database of complete *E. coli* genomes. The most closely related sequences were retrieved for *stx*_2a_-converting prophages from EHEC O157:H7 [46]; the high level of homology was confirmed by re-mapping of the sequencing reads to the O157:H7 prophage reference (Fig. 4). The presence of two DNA segments highly homologous to either O104:H4 or O157:H7 *stx*_2a_-converting prophages suggests a role of recombination in the evolution of type II prophages. This is in agreement with the view of recombination as the most common genetic event contributing to the mosaic structure and thus to the high genetic diversity of EHEC *stx*-converting phages [38, 41].

Remarkably, the type II prophages carry a single point mutation in the left half of their sequence, which is otherwise identical among type I, type II and O104 *stx*-converting prophages (Fig. 4). This mutation, which changes alanine (A) 273 into threonine (T) 273 in Stx2a A subunit (Fig. 2), is novel among the known diversity of Stx2 proteins and complements other mutations of A273 to amino acids with hydroxylated side chains (serine [S]and tyrosine [Y]- Additional file 5: Figure S2).

### Stx production and cytotoxicity of nEC strains

**Fig. 5:**
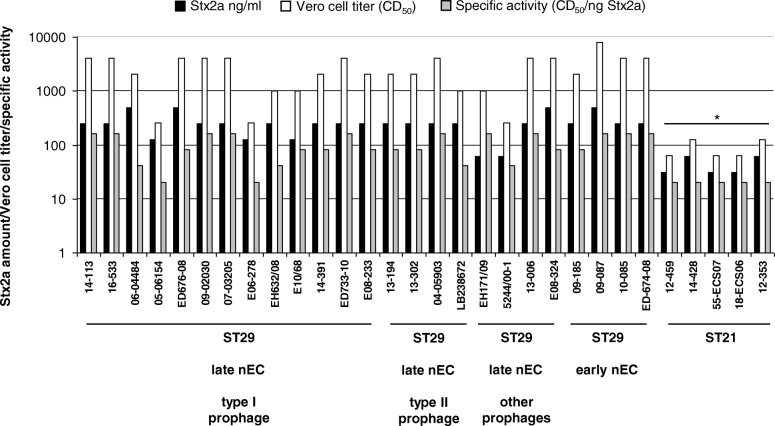
Stx2a production in nEC strains. The chart depicts Stx2a amounts, cytotoxicity titers, and specific activities of Stx2a produced by EHEC O26 ST29 late nEC strains harboring different types of *stx*_2a_-converting phages (as denoted in Fig. 2), ST29 early nEC strains and ST21 Stx2a-producing strains. The data are means from four measurements. *p < 0.01 for comparisons between the indicated characteristics of ST21 strains and ST29 strains (one-way ANOVA)

To determine whether or not the A273 → T273 mutation in Stx2a A subunit encoded by the type II *stx*_2a_-prophages influenced the toxicity of the resulting Stx2a protein, we compared the amounts, Vero cell cytotoxicity titers, and specific activities (CD_50_/ng toxin) of Stx2a produced by nEC strains harboring the type II prophages with those of strains harboring the type I or other *stx*_2a_-converting prophages. No significant differences were found (Fig. 5), indicating that the A273 → T273 mutation in the type II *stx*_2a_-prophage had no effect on Stx2a biological activity. Moreover, comparison of Stx2a amounts, cytotoxicity titers, and Stx2a specific activities between strains of the late nEC and early nEC did not reveal any differences in these characteristics within the ST29 nEC group (Fig. 5). However, all these characteristics of ST29 nEC strains were significantly higher than those of Stx2a-producing strains of ST21 clonal group (Fig. 5).

## Discussion

Using genome-wide SNP-based analysis, we demonstrate that EHEC O26 strains belonging to the nEC are not phylogenetically homogeneous, but consist of two distinct lineages. This finding extends the present knowledge about the clonal structure of these pathogens and confirms previous reports [9, 31, 35, 39] that *E. coli* O26:H11/H^−^ are, from the evolutionary point of view, highly dynamic with a potential for novel virulent clones to emerge rapidly. Based on their consecutive appearance and spread within Germany and the Czech Republic, we propose to term these lineages “early” nEC and “late” nEC, respectively. nEC was reported to have arisen in Germany during the 1990s [31, 33]; currently, both nEC lineages are widely distributed in Europe and were also isolated in Japan (Fig. 2), which highlights that nEC strains are capable of rapid spreading across countries and continents. Given the genetic distinctness of the early and late nEC, their expansions are likely to have occurred independently. Although the spread via routes common to other EHEC (i.e. via livestock and/or food trade) seems to be most likely, *E. coli* O26 corresponding to the nEC have only rarely been isolated from cattle and other animals [47, 48]. Moreover, none of 10 *E. coli* O26 nEC isolates (ST29C2 clade) present among 520 whole genome sequences analyzed by Ogura et al. [39], which represent a global population of human and animal *E. coli* O26, originated from cattle or other animals. Thus, reservoirs and possible ways of the worldwide spread of *E. coli* O26 nEC in general and of the late nEC in particular need to be further elucidated. This is particularly important because of the increasing frequency of the late nEC strains as causes of human diseases in some European countries during last years (Fig. 3). To create a basis for effective measures to control further spread of these pathogens, screening of ST29 *E. coli* O26 isolates from cattle and other animals, using the *sen/ent* SNP-based PCR developed in this study, combined with plasmid gene profiling might be a useful and simple tool for identification of strains of the early and late nEC. Altogether, the emergence of the late nEC both throughout and outside Europe supports a continuous evolution of *E. coli* O26:H11/H^−^ and a high propensity of new clones to spread [9, 35, 39].

Analysis of *stx*_2a_-converting prophages in nEC strains (Fig. 4) identified a predominant prophage (designated type I), which displayed a nearly complete sequence identity with the *stx*_2a_-converting prophages of EHEC O104:H4 strain, which caused the large devastating outbreak of HUS in Germany in 2011 [43]. Type I prophage is widespread across the nEC diversity (Fig. 2), implicating a single acquisition during early evolution of nEC (as also suggested in [38]). Based on available bibliographic data, the emergence of EHEC O26 nEC in the 1990s [31, 33] predates the isolation of Stx2a-producing EHEC O104:H4 [32, 43, 49], which was first isolated in 2001 [32]. This allows to speculate that EHEC O26 belonging to the nEC might have served as donors of *stx*_2a_-converting phages for enteroaggregative *E. coli* O104:H4 in the final step of evolution of the highly virulent, Stx2a-producing EHEC O104:H4 outbreak strain. Alternatively, EHEC O26 nEC and EHEC O104:H4 outbreak strain might have acquired their *stx*_2a_-converting phages independently from a common source. Beutin et al. [50] proposed that *stx*_2a_-converting prophages of EHEC O104:H4 might have originated from *stx*_2a_-converting phages present among bovine *E. coli* of various serotypes, with which they share nucleotide sequences of two specific DNA fragments, virion morphology, DNA restriction patterns, chromosomal integration site, and superinfection immunity [50]; however, this hypothesis needs to be confirmed by complete sequence analyses of the bovine phages.

Four late nEC strains were found to harbor a *stx*_2a_-converting prophage whose sequence consisted of two regions highly homologous to EHEC O104:H4 and EHEC O157:H7 *stx*_2a_-phages, respectively (designed type II; Fig. 4). These strains were scattered throughout the whole-genome phylogenetic tree and their most closely related strains harbored type I prophages (Fig. 2). This provides evidence that horizontal, rather than vertical pattern of type II prophages transfer was prevalent in late nEC evolution, thus suggesting an ongoing dissemination of type II phages in the *E. coli* O26 population. Notably, Stx2a encoded by type II prophages contain a novel amino acid change (A273 → T273). This mutation does not affect the potency of the toxin as demonstrated by its specific activity for Vero cells, which is similar to that of strains harboring type I prophages (Fig. 5). However, the localization of the mutation suggests a possible connection with Stx2a maturation. A273 follows immediately after arginine [R] 272; cleavage of Stx by the Golgi protease furin takes place precisely between these two residues [51, 52]. The cleavage generates the enzymatically active form of Stx2a A subunit [53]. The furin site where A273 binding takes place is a small hydrophilic pocket [54], which could explain the propensity of A273 for substitutions with hydroxyl-containing amino acids (Additional file 5: Figure S2). If there are any biological effects conferred by this Stx2a mutation specific for type II prophages, remains to be elucidated.

## Conclusions

Using genome-wide SNP-based analysis, this study presents the evidence of the split of the EHEC O26:H11/H^−^ nEC, which emerged in Germany in the 1990s and has spread throughout Europe [31], into two cryptic, yet distinct clones (early nEC and late nEC, Fig. 2). For EHEC O26, the combination of MLST, plasmid gene profiling and PCR-based *stx* subtyping has been regarded as sufficient for clone discrimination [9, 31]. The fact that strains of the early nEC and late nEC are indistinguishable by this approach (Fig. 1) emphasizes the necessity of using highly discriminatory methods such as the genome-wide SNP-based analysis to distinguish the most closely related epidemic lineages. For practical purposes, the SNP-based PCR developed in this study which targets a mutation in the *sen*/*ent* gene synapomorphic for the late nEC, represents a rapid and easy tool for distinguishing the early and late nEC strains in clinical microbiological laboratories and field studies. This will, in turn, enable further investigations of the geographic distribution of these pathogens, their clinical significance, and the epidemiology of human infections they cause.

Our results confirm that EHEC O26 nEC strains and *E. coli* O104:H4 German outbreak strain [43] share the same type of *stx*_2a_-converting prophage [38]. An additional *stx*_2a_-converting prophage (type II), present in a subset of the late nEC strains, displays several peculiarities (hybrid sequence, novel missense mutation in *stx*_2a_, multiple horizontal transmissions among late nEC) which warrant further investigation.

In summary, the discriminatory power of whole genome sequencing allows for accurate reconstruction of evolutionary events which accompanied the emergence of novel clonal lineages of EHEC O26.

## Methods

### Database mining

*Escherichia coli* complete or draft genomic sequences were retrieved from the GenBank database [55] on January 31st, 2017. The precompiled genome neighbors of *E. coli* strain St. Olav179 (a member of late nEC, accession JZED00000000 [56]) were extracted. Strains with symmetric identity below 92.0% were discarded since they typically represented serotypes other than O26. Among remaining strains, those belonging to non-O26 serotypes were discarded. In addition, genomic sequences of strains included in later studies which focused on genomic evolution of *E. coli* O26 [9, 38] were included in the dataset. In total, 127 strains from public sources were collected for genomic comparisons (Additional file 1: Table S1).

### Whole-genome sequencing

Sixteen EHEC O26:H11/H^−^ strains were isolated from patients with HUS (*n* = 10) or with bloody (*n* = 4) or non-bloody (*n* = 2) diarrhea without HUS in the Czech Republic between 2006 and 2016. Additional 16 EHEC O26:H11/H^−^ nEC strains sequenced in this study originated from Germany (*n* = 11), Italy (*n* = 3), and Austria (*n* = 2) and were isolated from patients with HUS (*n* = 10) or non-bloody diarrhea (*n* = 6) (Additional file 1: Table S1). The Czech and German strains were from the authors´ laboratories. The Italian and Austrian isolates were kindly provided by Stefano Morabito (European Union Reference Laboratory for *E. coli*, Instituto Superiore di Sanità, Rome, Italy) and Dorothea Orth-Höller (Division of Hygiene and Medical Microbiology, Innsbruck Medical University, Austria), respectively. WGS was performed as described previously [57]. Briefly, after inoculation of a single colony into nutrient broth (Heipha, Eppelheim, Germany) and overnight incubation (37 °C), genomic DNA was extracted using a MagAttract HMW DNA kit (Qiagen, Hilden, Germany) with the addition of lysozyme (Sigma-Aldrich, Taufkirchen, Germany) following the manufacturer’s instructions. Subsequently, the sequencing libraries were prepared from the genomic DNA using the Nextera sample preparation kit (Illumina, Inc., San Diego, CA, USA) for a 150-bp or 250-bp paired-end sequencing run on a single MiSeq instrument (Illumina) in accordance with the manufacturer’s recommendations. Libraries were scaled to reach 100-fold sequencing coverage. Subsequent quality trimming and de novo assembly were performed using the default parameters of CLC Genomics Workbench software (CLC bio, Arhus, Denmark) and the CLC Genomics Workbench de novo assembler (CLC bio). The draft genomes of strains sequenced in our study were annotated using the NCBI prokaryotic genome annotation pipeline [58] and are available in the GenBank under the accession numbers listed in Additional file 1: Table S1.

### Bioinformatic analysis

#### Phylogeny reconstruction

Genome-wide SNP-based phylogeny was chosen for robust inference of *E. coli* O26 evolutionary history. Genomic sequences were uploaded to the CSIPhylogeny v1.4 website (https://cge.cbs.dtu.dk/services/CSIPhylogeny/) and automatically processed with default settings. SNP analysis was carried out using a set of algorithms as described in [59]; FastTree [60] was used for phylogram construction. Phylogenetic trees were visualized using MEGA7 [61]. Genomic sequences of *E. coli* O111:H^−^ strain 11128 and *E. coli* O26:H11 strain 11368 were included as reference genomes for EHEC O26 and nEC SNP-based phylogeny, respectively.

#### In silico analysis of molecular diagnostic markers

The presence of pathogenicity-related genes (plasmid genes EHEC-*hlyA*, *katP*, *espP* and *etpD*, Shiga toxin genes *stx*_1a_ and *stx*_2a_) was assessed using TBLASTN [62] integrated in Geneious R9.1 [63], using protein query sequences from *E. coli* O26 strain 11368 (GenBank accession: AP010953) or *E. coli* O157 strain Sakai (GenBank accession: NC_002695). Presence or absence and identity of encoded proteins with respect to query sequences are listed in Additional file 1: Table S1.

#### Synapomorphic SNPs determination

Genomic sequences of several strains representing the genomic diversity of *E. coli* O26 (early nEC: St. Olav174, FHI24, FHI27; late nEC: ED676, St. Olav179, 36708, STEC931; ST21: ED180, ED729, STEC1117, CVM9942, non-nEC ST29: C792_92, CFSAN025102, 200C-3689, 34827) were aligned with Progressive MAUVE [64]. Presumptive synapomorphic SNPs (i.e. SNPs specific for early nEC, late nEC or nEC in general) were manually identified among the SNPs reported by MAUVE intergenomic comparison tool, based on their exclusive occurrence in corresponding lineages. For the definite validation, BLAST search [65] was performed against all genomic sequences included in the study (Additional file 1: Table S1); SNPs were considered synapomorphic if they were present in all members of a particular lineage and absent in all remaining O26 strains (Additional file 4: Table S2).

#### Stx-converting phage analysis

The paired-end sequencing reads were mapped onto *stx*_2a_-converting prophage sequences present in *E. coli* O104:H4 strain C227–11 (GenBank accession: CP011331) and *E. coli* O157:H7 strain Sakai (GenBank accession: NC_002695) using Geneious R9 platform [63]. The mapping setting was as follows: max. 20% gaps per read, max. 10% mismatches per read. SNPs (true mutations) were called with coverage ≥5 and frequency ≥ 90% (with respect to the reference). Variable sites were called where a mixture of bases was present in assembly of sequencing reads (coverage ≥5, frequency of minor variant ≥30%).

### Late nEC-discriminating PCR

A SNP-based PCR was used as a quick screening method to detect a missense mutation in the *sen*/*ent* gene (encoding *Shigella flexneri* enterotoxin 2), specific for late nEC (102S → Y; Additional file 4: Table S2). The following primers were designed using Primer/BLAST [66]: forward primer sen-F (5´-TCTAAATGGAAAGGTTAGTGATTGC-3′) and reverse primer sen-RG (5´-GGTTATATATAACGCTTCCCCAAG-3′). In sen-RG, the 3′-terminal guanine is not complementary to the late nEC-specific SNP. The predicted amplicon length was 233 bp. PCR reactions were performed in CFX96 Touch PCR Detection System (Bio-Rad) using reagents from Top-Bio (Czech Republic), primers obtained from Generi Biotech (Czech Republic) and bacterial lysates (boiling method) as DNA templates. PCR conditions were: 94 °C for 5 min, followed by 30 cycles of denaturation (94 °C for 30 s), annealing (53 °C for 60 s), and extension (72 °C for 60 s) and a final extension step at 72 °C for 5 min. One early nEC and one late nEC strain were used as controls in each PCR. After electrophoretic separation on agarose gel and ethidium bromide staining, early nEC strains produced strong amplicons, while late nEC strains displayed substantially weaker amplicons due to a reduced pairing of 3′ guanine with template DNA containing the SNP (Additional file 3: Table S3). All nEC strains analyzed by WGS in this study were tested with the late nEC-discriminating PCR; 100% correlation between the *sen*/*ent*-specific PCR and WGS results was achieved (Additional file 3: Table S3).

### Detection of Stx production and cytotoxicity

#### VTEC RPLA

VTEC RPLA (Verotoxin-producing *E. coli* reverse passive latex agglutination) assay (Denka Seiken Company, Ltd., Tokyo, Japan) was performed, according to the manufacturer’s instructions, with serially diluted sterile supernatants prepared from overnight shaken broth cultures by centrifugation (15 min, 4000 rpm, 4 °C) and subsequent filtration through 0.22 μm syringe filters (P-LAB, Prague, Czech Republic). Stx1a and Stx2a titers were expressed as reciprocals of the supernatants´ dilutions that produced a clear agglutination of latex particles sensitized with anti-Stx1a and anti-Stx2a antibody, respectively. Stx1a and Stx2a in the supernatants were quantified by comparison of titers produced by the supernatants with those of Stx2a (VT2) and Stx1a (VT1) standards with known toxin concentrations.

#### Vero cell assay

Vero-B4 cells (ACC-33; German collection of microorganisms and cell cultures, Braunschweig, Germany) were maintained in Dulbecco’s modified Eagle medium (DMEM) with 4.5 g/l of glucose and glutamine (Lonza, Cologne, Germany) supplemented with 10% of fetal bovine serum (Sigma-Aldrich, Prague, Czech Republic). In the cytotoxicity assay [67], semiconfluent Vero cell monolayers grown in 96-well plates were incubated with two-fold dilutions of supernatants prepared as above for 72 h. After removing medium with detached cells, remnant adherent cells were fixed (2% formalin), stained (0.13% crystal violet), washed, and after eluting of crystal violet with 50% ethanol, absorbance (OD_570_) was measured by FLUOstar Omega (BMG Labtech, Ortenberg, Germany). Cytotoxicity titers were expressed as reciprocal values of the sample dilutions that killed 50% of cells (CD_50_). Specific activities of Stx in supernatants were expressed as CD_50_/ng of Stx.

#### Statistical analysis

Data on Stx2a amounts, cytotoxicity titers, and specific activities of Stx2a produced by different groups of strains were analyzed with one-way analysis of variance (ANOVA). *p* values < 0.05 were considered significant.

## Additional files

Additional file 1: **Table S1.** Summary information about 159 *E. coli* O26 isolates whose genomic sequences were included for comparative analysis. Presence/absence of molecular diagnostic markers and their identity with query sequences are indicated. (DOCX 144 kb)

Additional file 2: **Figure S1.** Core genome-derived phylogeny of 159 *E. coli* O26 isolates. The phylogram (inferred using the CSIPhylogeny pipeline [59]) represents the full version of contracted phylogram presented in Fig. 1. (PDF 32 kb)

Additional file 3: **Table S3.** Discrimination of early nEC and late nEC strains by *sen*/*ent* SNP-specific PCR, WGS and PFGE. All nEC strains characterized in this study by WGS (Fig. 2) and/or by *sen*/*ent* SNP-specific PCR (Fig. 3) are listed, together with results obtained by either method and PFGE clustering [31] (n.a., not available). Representative gel image which demonstrates the performance of the *sen*/*ent* SNP-specific PCR in differentiating early- and late nEC strains is included. (XLSX 69 kb)

Additional file 4: **Table S2.** SNPs synapomorphic for the early nEC, late nEC or nEC as whole. Missense mutations in fimbrial proteins are denoted in bold. (XLS 112 kb)

Additional file 5: **Figure S2.** Alignment of all unique Stx2 protein sequences present in the GenBank. Residue A273 is marked with a red rectangle on the top of alignment. (PDF 6180 kb)
