# Supplementary material for: Attack of the clones: whole genome-based characterization of two closely related enterohemorrhagic Escherichia coli O26 epidemic lineages
Source: BMC Genomics. 2018 Aug 31;19:647. doi: 10.1186/s12864-018-5045-7 (PMC6119250; doi:10.1186/s12864-018-5045-7)
Supplement: Supplementary file 1 — Table S1. Summary information about 159 E. coli O26 isolates whose genomic sequences were included for comparative analysis. Presence/absence of molecular diagnostic markers and their identity with query sequences are indicated. (DOCX 144 kb) [file 12864_2018_5045_MOESM1_ESM.docx]

|  |  |  |  |  |  |  | **Molecular diagnostic markers**  *tblastn results using protein query sequences as indicated in column headings; WT = 100% identity* | | | | | | | |  |
| --- | --- | --- | --- | --- | --- | --- | --- | --- | --- | --- | --- | --- | --- | --- | --- |
|  |  |  |  |  |  |  | **Plasmid genes** | | | | **Stx1a** | | **Stx2a** | |  |
| **Strain** | **WGS assembly accession (source)** | **Clade [1]** | **Serotype** | **Country of isolation** | **Year of isolation** | **Source material or condition**  **(if specified)** | **EHEC-HlyA (O26:H11 str. 11368)** | **KatP (O26:H11 str. 11368)** | **EspP (O157:H7 str. Sakai)** | **EtpD (O157:H7 str. Sakai)** | **Stx1a subunit A (O26:H11 str. 11368)** | **Stx1a subunit B**  **(O26:H11 str. 11368)** | **Stx2a subunit A (O157:H7 str. Sakai)** | **Stx2a subunit B**  **(O157:H7 str. Sakai)** | **Reference** |
| **11368** | GCA_000091005.1 | ST21 | O26:H11 | Japan | 2001 | D | WT | WT | x | x | WT | WT | x | x | [2] |
| **06-3464** | GCA_000622465.2 | ST21 | O26:H11 | USA | not specified |  | WT | WT | WT | x | x | WT | x | x | [3] |
| **03-3500** | GCA_000622445.2 | ST21 | O26:H11 | USA | not specified |  | WT | WT | WT | x | WT | WT | x | x | [3] |
| **05-3646** | GCA_000617065.1 | ST21 | O26:H11 | USA | not specified |  | WT | x | x | x | WT | WT | x | x | [3] |
| **05-6544** | GCA_001309465.1 | ST21 | O26:H11 | Canada | 2014 |  | x | x | x | x | WT | WT | x | x | [4] |
| **2009C-3996** | GCA_000622595.2 | ST21 | O26:H11 | USA | 2009 |  | WT | WT | WT | x | WT | WT | x | x | [3] |
| **2009C-4747** | GCA_000632575.1 | ST21 | O26:H11 | USA | 2009 |  | WT | WT | WT | x | WT | WT | x | x | [3] |
| **2009C-4760** | GCA_000622615.2 | ST21 | O26:H11 | USA | 2009 |  | WT | WT | WT | x | WT | WT | x | x | [3] |
| **2009C-4826** | GCA_000622635.2 | ST21 | O26:H11 | USA | 2009 |  | WT | WT | WT | x | WT | WT | x | x | [3] |
| **2010C-3051** | GCA_000622675.2 | ST21 | O26:H11 | USA | 2010 |  | WT | WT | WT | x | WT | WT | x | x | [3] |
| **2010C-3472** | GCA_000622695.2 | ST21 | O26:H11 | USA | 2010 |  | WT | WT | WT | x | WT | WT | x | x | [3] |
| **2010C-3871** | GCA_000622715.2 | ST21 | O26:H11 | USA | 2010 |  | WT | WT | WT | x | WT | WT | incomplete | WT | [3] |
| **2010C-3902** | GCA_000622735.2 | ST21 | O26:H11 | USA | 2010 |  | WT | WT | WT | x | WT | WT | WT | WT | [3] |
| **2010C-4244** | GCA_000622575.2 | ST21 | O26:H11 | USA | 2010 |  | WT | WT | WT | x | WT | WT | x | x | [3] |
| **2010C-4347** | GCA_000614845.2 | ST21 | O26:H11 | USA | 2010 |  | WT | WT | WT | x | WT | WT | x | x | [3] |
| **2010C-4430** | GCA_000622505.2 | ST21 | O26:H11 | USA | 2010 |  | WT | WT | WT | x | x | WT | x | x | [3] |
| **2010C-4788** | GCA_000617505.2 | ST21 | O26:NM | USA | 2010 |  | WT | WT | WT | x | WT | WT | x | x | [3] |
| **2010C-4819** | GCA_000622485.2 | ST21 | O26:H11 | USA | 2010 |  | WT | WT | WT | x | WT | WT | WT | WT | [3] |
| **2010C-4834** | GCA_000622655.2 | ST21 | O26:H11 | USA | 2010 |  | 553_Y→F | WT | WT | x | WT | WT | x | x | [3] |
| **2010C-5028** | GCA_000622535.2 | ST21 | O26:H11 | USA | 2010 |  | WT | WT | WT | x | WT | WT | x | x | [3] |
| **2010EL-1699** | GCA_000622555.1 | ST21 | O26:H11 | USA | 2010 |  | WT | WT | WT | x | WT | WT | x | x | [3] |
| **2011C-3270** | GCA_000622775.2 | ST21 | O26:H11 | USA | 2011 |  | WT | WT | WT | x | WT | WT | x | x | [3] |
| **2011C-3274** | GCA_000703325.1 | ST21 | O26:H11 | USA | 2011 |  | WT | WT | 100_V→A | x | WT | WT | x | x | [3] |
| **2011C-3282** | GCA_000622795.2 | ST21 | O26:H11 | USA | 2011 |  | WT | WT | WT | x | WT | WT | x | x | [3] |
| **2011C-3387** | GCA_000622815.1 | ST21 | O26:H11 | USA | 2011 |  | WT | WT | WT | x | WT | WT | x | x | [3] |
| **2011C-3506** | GCA_000622835.1 | ST21 | O26:H11 | USA | 2011 |  | WT | WT | WT | x | WT | WT | x | x | [3] |
| **2011C-3655** | GCA_000622855.2 | ST21 | O26:H11 | USA | 2011 |  | WT | WT | WT | x | WT | WT | x | x | [3] |
| **304-ECS08** | QFRY00000000 | ST21 | O26:H11 | Czech Republic | 2008 | D | WT | WT | WT | x | WT | WT | x | x | this work |
| **900105_(10e)** | GCA_000194725.2 | ST21 | O26:H11 | USA | 1990 |  | x | x | x | x | WT | WT | x | x | https://www.ncbi.nlm.nih.gov/bioproject/PRJNA51137 |
| **97-3250** | GCA_000695115.1 | ST21 | O26:H11 | USA | 1997 |  | WT | WT | WT | x | WT | x | WT | WT | https://www.ncbi.nlm.nih.gov/bioproject/PRJNA242431 |
| **ATCC_BAA-2196** | GCA_000508365.1 | ST21 | O26:H11 | USA | 2003 |  | WT | WT | WT | x | WT | WT | WT | WT | https://www.ncbi.nlm.nih.gov/bioproject/PRJNA221245/ |
| **C155_11** | GCA_000446155.2 | ST21 | not specified | Denmark | not specified | D | WT | WT | WT | x | x | x | x | x | [5] |
| **CFSAN001629** | GCA_000313405.1 | ST21 | O26:H11 | USA | 1997 |  | x | x | x | x | WT | WT | x | x | [6] |
| **CFSAN025090** | GCA_001571495.1 | ST21 | O26:H11 | USA | 2011 |  | WT | x | WT | x | WT | WT | x | x | [6] |
| **CFSAN025091** | GCA_001571485.1 | ST21 | O26:H11 | USA | 2011 |  | WT | x | WT | x | WT | WT | x | x | [6] |
| **CFSAN033951** | GCA_001696245.1 | ST21 | O26:H11 | Canada | 1977 |  | WT | WT | WT | x | WT | WT | x | x | https://www.ncbi.nlm.nih.gov/bioproject/PRJNA230969/ |
| **CVM10021** | GCA_000276945.1 | ST21 | O26:H11 | not specified | 1995 |  | x | WT | WT | x | WT | WT | x | x | [7] |
| **CVM10026** | GCA_000264035.1 | ST21 | O26:H11 | not specified | 1955 |  | WT | x | x | x | WT | WT | x | x | [7] |
| **CVM10030** | GCA_000276845.1 | ST21 | O26:H11 | not specified | 1995 |  | WT | x | x | x | WT | WT | x | x | [7] |
| **CVM10224** | GCA_000276785.1 | ST21 | O26:H11 | not specified | 1997 |  | x | x | x | x | WT | WT | x | x | [7] |
| **CVM9942** | GCA_000264015.1 | ST21 | O26:H11 | not specified | 1983 |  | x | x | x | x | WT | WT | x | x | [7] |
| **CVM9952** | GCA_000276885.1 | ST21 | O26:H11 | not specified | 1985 |  | x | x | x | x | WT | WT | x | x | [7] |
| **DEC10A** | GCA_000249995.2 | ST21 | O26:H11 | USA | 2013 |  | x | x | x | x | WT | WT | x | x | [8] |
| **DEC10B** | GCA_000250015.2 | ST21 | O26:H11 | USA | 2013 |  | x | WT | x | x | WT | WT | x | x | [8] |
| **DEC10C** | GCA_000250035.2 | ST21 | O26:H11 | USA | 2013 |  | x | x | x | x | WT | WT | x | x | [8] |
| **DEC8D** | GCA_000249855.2 | ST21 | O26:H11 | USA | 2013 |  | WT | WT | WT | x | x | x | x | x | [8] |
| **EC1865** | GCA_000303395.2 | ST21 | not specified | USA | 2007 |  | WT | x | WT | x | WT | WT | x | x | https://www.ncbi.nlm.nih.gov/bioproject/PRJNA65969/ |
| **ED075** | GCA_900015795.1 | ST21 | O26 | Italy | 1995 | D | WT | WT | WT | x | WT | WT | x | x | [9] |
| **ED195** | GCA_900008835.1 | ST21 | O26 | Italy | 1995 | D | WT | WT | WT | x | WT | WT | x | x | [9] |
| **ED392** | GCA_900010065.1 | ST21 | O26 | Italy | 1988 | D | WT | WT | WT | x | WT | WT | x | x | [9] |
| **ED423** | GCA_900008845.1 | ST21 | O26 | Italy | 1999 | D | WT | WT | 1037_G→D | x | WT | WT | x | x | [9] |
| **ED669** | GCA_900014215.1 | ST21 | O26 | Italy | 2008 | D | WT | WT | WT | x | WT | 21_T→M | x | x | [9] |
| **ED729** | GCA_900015885.1 | ST21 | O26 | Italy | 2010 | D | WT | x | WT | x | WT | WT | x | x | [9] |
| **EPECa14** | GCA_000188755.2 | ST21 | O26:NM | Brazil | 1999 | D | 5 mutations | WT | x | x | WT | WT | x | x | https://www.ncbi.nlm.nih.gov/bioproject/PRJNA40267/ |
| **ESC172** | GCA_001660325.1 | ST21 | not specified | Japan | not specified |  | WT | WT | WT | x | WT | WT | x | x | https://www.ncbi.nlm.nih.gov/bioproject/PRJNA286916/ |
| **ESC480** | GCA_001660335.1 | ST21 | not specified | Japan | not specified |  | WT | WT | WT | x | WT | WT | x | x | https://www.ncbi.nlm.nih.gov/bioproject/PRJNA286916/ |
| **FHI1** | GCA_000752435.1 | ST21 | O26 | Norway | 2009 | D | WT | WT | WT | x | WT | WT | x | x | [10] |
| **FHI20** | GCA_000940115.1 | ST21 | O26 | Norway | 2006 | D | WT | WT | WT | x | WT | WT | x | x | [10] |
| **FHI3** | GCA_000752195.1 | ST21 | O26 | Norway | 2002 | D | WT | x | x | x | x | x | WT | WT | [10] |
| **FHI4** | GCA_000951835.2 | ST21 | O26 | Norway | 2002 | HUS | WT | x | WT | x | x | x | WT | WT | [10] |
| **FHI46** | GCA_000752115.1 | ST21 | O26 | Norway | 2009 | D | WT | WT | WT | x | WT | WT | x | x | [10] |
| **FHI47** | GCA_000752655.1 | ST21 | O26 | Norway | 2009 | BD | WT | WT | WT | x | WT | WT | x | x | [10] |
| **FHI50** | GCA_000752355.1 | ST21 | O26 | Norway | 2009 | BD | WT | WT | WT | x | WT | WT | x | x | [10] |
| **FHI60** | GCA_000752255.1 | ST21 | O26 | Norway | 2009 | BD | WT | WT | x | x | WT | WT | x | x | [10] |
| **FHI61** | GCA_000752515.1 | ST21 | O26 | Norway | 2009 | D | WT | WT | x | x | WT | WT | x | x | [10] |
| **FHI70** | GCA_000940655.1 | ST21 | O26 | Norway | 2010 | D | WT | WT | x | x | WT | WT | x | x | [10] |
| **FHI77** | GCA_000752915.1 | ST21 | O26 | Norway | 2010 | BD | WT | WT | x | x | WT | WT | x | x | [10] |
| **FHI78** | GCA_000752295.1 | ST21 | O26 | Norway | 2010 | BD | WT | WT | x | x | WT | WT | x | x | [10] |
| **FHI90** | GCA_000936085.2 | ST21 | O26 | Norway | 2010 | D | WT | WT | WT | x | WT | WT | x | x | [10] |
| **FORC_028** | GCA_001721125.1 | ST21 | not specified | South Korea | 2003 | D | x | x | x | x | WT | WT | x | x | https://www.ncbi.nlm.nih.gov/bioproject/PRJNA294502/ |
| **OLC-464** | GCA_000948745.1 | ST21 | O26:H11 | Canada | 1995 |  | WT | WT | WT | x | WT | WT | x | x | [11] |
| **STEC_1117** | GCA_001607095.1 | ST21 | O26:H11 | Netherlands | 2013 | D | WT | WT | WT | x | WT | WT | x | x | [12] |
| **STEC_1236** | GCA_001607205.1 | ST21 | O26:H11 | Netherlands | 2013 | D | WT | WT | WT | x | WT | WT | x | x | [12] |
| **STEC_2110.3** | GCA_001606975.1 | ST21 | O26:H11 | Netherlands | 2013 | stool | WT | WT | WT | x | x | x | x | x | [12] |
| **STEC_2144** | GCA_001606985.1 | ST21 | O26:H11 | Netherlands | 2013 | D | WT | WT | WT | x | WT | WT | x | x | [12] |
| **STEC_2346** | GCA_001607255.1 | ST21 | O26:H11 | Netherlands | 2013 | D | WT | WT | WT | x | WT | WT | x | x | [12] |
| **STEC_2920** | GCA_001607875.1 | ST21 | O26:H11 | Netherlands | 2013 | BD | WT | x | WT | x | WT | WT | x | x | [12] |
| **STEC_380** | GCA_001608015.1 | ST21 | O26:H11 | Netherlands | 2013 | D | WT | WT | WT | x | WT | WT | x | x | [12] |
| **STEC_477** | GCA_001607015.1 | ST21 | O26:H11 | Netherlands | 2013 | D | WT | WT | WT | x | WT | WT | x | x | [12] |
| **STEC_479** | GCA_001607025.1 | ST21 | O26:H11 | Netherlands | 2013 | BD | WT | WT | WT | x | WT | WT | x | x | [12] |
| **STEC_487** | GCA_001609765.1 | ST21 | O26:H11 | Netherlands | 2013 | D | WT | WT | WT | x | WT | WT | x | x | [12] |
| **STEC_709** | GCA_001606365.1 | ST21 | O26:H11 | Netherlands | 2013 | BD | WT | WT | WT | x | WT | WT | 273_A→T | x | [12] |
| **STEC_764** | GCA_001608075.1 | ST21 | O26:H11 | Netherlands | 2013 | D | WT | x | WT | x | WT | WT | x | x | [12] |
| **STEC_563** | GCA_001609855.1 | ST21 | O26:H11 | netherlands | 2013 | D | WT | WT | WT | x | WT | WT | 124_G→D | WT | [12] |
| **STEC_1293** | GCA_001606815.1 | ST21 | O26:H11 | Netherlands | 2013 | BD | WT | WT | WT | x | WT | WT | 124_G→D | WT | [12] |
| **OLC-731** | GCA_000948815.1 | ST21 | O26:H11 | Canada | 2007 |  | WT | WT | WT | x | WT | WT | 124_G→D | 13_A→V | [11] |
| **18-ECS06** | QFRZ00000000 | ST21 | O26:NM | Czech Republic | 2006 | BD,HUS | WT | WT | WT | x | x | x | 124_G→D | 13_A→V | this work |
| **12-459** | QFSA00000000 | ST21 | O26 | Czech Republic | 2012 | BD | WT | WT | WT | x | x | x | 124_G→D | 13_A→V | this work |
| **14-428** | QFSB00000000 | ST21 | O26:NM | Czech Republic | 2014 | D, HUS | WT | WT | WT | x | x | x | 124_G→D | 13_A→V | this work |
| **ED180** | GCA_900015855.1 | ST21 | O26 | Italy | 1955 | D | WT | WT | WT | x | x | x | 124_G→D | 13_A→V | [9] |
| **55-ECS07** | QFSC00000000 | ST21 | O26:NM | Czech Republic | 2007 | D, HUS | WT | WT | WT | x | x | x | 124_G→D | 13_A→V | this work |
| **DEC10D** | GCA_000250055.2 | ST21 | O26:H11 | USA | 2013 |  | x | x | 115_T→I | x | x | x | x | x | [8] |
| **36084** | GCA_001039075.1 | ST21 | O26:H11 | France | 2013 | HUS | WT | WT | 115_T→I | x | x | x | 124_G→D | 13_A→V | [13] |
| **36079** | GCA_001039125.2 | ST21 | O26:H11 | France | 2013 | HUS | WT | WT | 1222_T→I | x | x | x | 124_G→D | 13_A→V | [14] |
| **12-353** | QFSD00000000 | ST21 | O26:H11 | Czech Republic | 2012 | BD, HUS-lethal | WT | WT | 1222_T→I | x | x | x | 124_G→D | WT | this work |
| **FHI2** | GCA_000752495.1 | ST29C2 | O26 | Norway | 2002 | D | x | x | x | 9 mutations | x | x | x | x | [10] |
| **15-496** | QFSE00000000 | ST29C2 | O26 | Czech Republic | 2015 | D | 349_A→S | x | x | 9 mutations | Stx1c (WT) | Stx1c (WT) | x | x | this work |
| **09-087** | QFSF00000000 | ST29C2 | O26:H11 | Czech Republic | 2009 | D, HUS-lethal | 349_A→S | x | x | 9 mutations | x | x | WT | WT | this work |
| **09-185** | QFSG00000000 | ST29C2 | O26:NM | Czech Republic | 2009 | D, HUS | 349_A→S | x | x | 9 mutations | x | x | WT | WT | this work |
| **10-085** | QFSH00000000 | ST29C2 | O26:NM | Czech Republic | 2010 | D, HUS | 349_A→S | x | x | 9 mutations | x | x | WT | WT | this work |
| **2602-4** | Dr. Iyoda | ST29C2 | O26:H11 | Japan | 2012 | HUS | 349_A→S | x | x | 9 mutations | x | x | WT | WT | [1] |
| **2602-7** | Dr. Iyoda | ST29C2 | O26:H11 | Japan | 2013 | BD | 349_A→S | x | x | 9 mutations | x | x | WT | WT | [1] |
| **ED_674-08** | QFSI00000000 | ST29C2 | O26:H11 | Italy | 2008 | HUS | 349_A→S | x | x | 9 mutations | x | x | WT | WT | this work |
| **ED411** | GCA_900015865.1 | ST29C2 | O26 | Italy | 1999 | D | 349_A→S | x | x | 9 mutations | x | x | WT | WT | [9] |
| **FHI24** | GCA_000936225.2 | ST29C2 | O26 | Norway | 2001 | D | 349_A→S | x | x | 9 mutations | x | x | WT | WT | [10] |
| **FHI27** | GCA_000951875.1 | ST29C2 | O26 | Norway | 2008 | D | 349_A→S | x | x | 9 mutations | x | x | WT | WT | [10] |
| **FHI36** | GCA_000753215.1 | ST29C2 | O26 | Norway | 2009 | D | 349_A→S | x | x | 9 mutations | x | x | WT | WT | [10] |
| **FHI39** | GCA_000752875.1 | ST29C2 | O26 | Norway | 2009 | D | 349_A→S | x | x | 9 mutations | x | x | WT | WT | [10] |
| **FHI79** | GCA_000939955.1 | ST29C2 | O26 | Norway | 2010 | D | 349_A→S | x | x | 9 mutations | x | x | WT | WT | [10] |
| **St._Olav174** | GCA_000965665.1 | ST29C2 | O26 | Norway | 2013 | D | 349_A→S | x | x | 9 mutations | x | x | WT | WT | [15] |
| **36708** | GCA_001039135.1 | ST29C2 | O26:H11 | France | 2013 | HUS/stool | 349_A→S | x | x | 9 mutations | x | x | WT | WT | [13] |
| **04-05903** | QFSJ00000000 | ST29C2 | O26:H11 | Germany | 2004 | HUS | 349_A→S | x | x | 9 mutations | x | x | 273_A→T | WT | this work |
| **05-06154** | QFSK00000000 | ST29C2 | O26:H11 | Germany | 2005 | D | 349_A→S | x | x | 9 mutations | x | x | WT | WT | this work |
| **06-04484** | QFSL00000000 | ST29C2 | O26:H11 | Germany | 2006 | D | 349_A→S | x | x | 9 mutations | x | x | WT | WT | this work |
| **07-03205** | QFSM00000000 | ST29C2 | O26:H11 | Germany | 2007 | HUS | 349_A→S | x | x | 9 mutations | x | x | WT | WT | this work |
| **09-02030** | QFSN00000000 | ST29C2 | O26:H11 | Germany | 2009 | D | 349_A→S | x | x | 9 mutations | x | x | WT | WT | this work |
| **13-006** | QFSO00000000 | ST29C2 | O26:H11 | Czech Republic | 2013 | HUS | 349_A→S | x | x | 9 mutations | x | x | WT | WT | this work |
| **13-194** | QFSP00000000 | ST29C2 | O26:H11 | Czech Republic | 2013 | D, HUS | 349_A→S | x | x | 9 mutations | x | x | 273_A→T | WT | this work |
| **13-302** | QFSQ00000000 | ST29C2 | O26:H11 | Czech Republic | 2013 | D, HUS | 349_A→S | x | x | 9 mutations | x | x | 273_A→T | WT | this work |
| **14-113** | QFSR00000000 | ST29C2 | O26 | Czech Republic | 2014 | D | 349_A→S | x | x | 9 mutations | x | x | WT | WT | this work |
| **14-391** | QFSS00000000 | ST29C2 | O26 | Czech Republic | 2014 | D | 349_A→S | x | x | 9 mutations | x | x | WT | WT | this work |
| **16-533** | QFST00000000 | ST29C2 | O26 | Czech Republic | 2016 | BD | 349_A→S | x | x | 9 mutations | x | x | WT | WT | this work |
| **2602-5** | Dr. Iyoda | ST29C2 | O26:H11 | Japan | 2008 | BD | 349_A→S | x | x | 9 mutations | x | x | WT | WT | [1] |
| **5244-00-1** | QFSU00000000 | ST29C2 | O26:H11 | Germany | 2000 | HUS | 349_A→S | x | x | 9 mutations | x | x | WT | WT | this work |
| **E-06-278** | QFSV00000000 | ST29C2 | O26:H11 | Germany | 2006 | HUS | 349_A→S | x | x | 9 mutations | x | x | WT | WT | this work |
| **E-08-233** | QFSW00000000 | ST29C2 | O26:H11 | Germany | 2008 | HUS | 349_A→S | x | x | 9 mutations | x | x | WT | WT | this work |
| **E-08-324** | QFSX00000000 | ST29C2 | O26:H11 | Germany | 2008 | D | 349_A→S | x | x | 9 mutations | x | x | WT | WT | this work |
| **E-10-68** | QFSY00000000 | ST29C2 | O26:H+ | Germany | 2010 | HUS | 349_A→S | x | x | 9 mutations | x | x | WT | WT | this work |
| **ED_676-08** | QFSZ00000000 | ST29C2 | O26:H11 | Italy | 2008 | HUS | 349_A→S | x | x | 9 mutations | x | x | WT | WT | this work |
| **ED_733-10** | QFTA00000000 | ST29C2 | O26:H11 | Italy | 2010 | HUS | 349_A→S | x | x | 9 mutations | x | x | WT | WT | this work |
| **ED676** | GCA_900015385.1 | ST29C2 | O26 | Italy | 2008 | D | 349_A→S | x | x | 9 mutations | x | x | WT | WT | [1] |
| **EH_171-09** | QFTB00000000 | ST29C2 | O26:H11 | Austria | 2009 | D | 349_A→S | x | x | 9 mutations | x | x | WT | WT | this work |
| **EH_632-08** | QFTC00000000 | ST29C2 | O26:H11 | Austria | 2008 | D | 349_A→S | x | x | 9 mutations | x | x | WT | WT | this work |
| **LB238672** | QFTD00000000 | ST29C2 | O26:H11 | Germany | 2012 | HUS | 349_A→S | x | x | 9 mutations | x | x | 273_A→T | WT | this work |
| [**St._Olav179**](https://www.ncbi.nlm.nih.gov/genome/167?genome_assembly_id=228867) | [GCA_000965705.1](https://www.ncbi.nlm.nih.gov/assembly/GCA_000965705.1) | ST29C2 | O26 | Norway | 2014 |  | 349_A→S | x | x | 9 mutations | x | x | WT | WT | [15] |
| **STEC_931** | GCA_001607075.1 | ST29C2 | O26:H11 | Netherlands | 2013 | D | 349_A→S | x | x | 9 mutations | x | x | WT | WT | [12] |
| **C792_92** | GCA_000446885.2 | no assigned | not specified | Italy | not specified |  | x | x | x | 9 mutations | x | x | x | x | [5] |
| **2009C-3612** | GCA_000622875.2 | ST29C1 | O26:H11 | USA | 2009 |  | 4 mutations | x | 22 mutations | x | x | x | WT | WT | [3] |
| **2009C-3689** | GCA_000622755.2 | ST29C1 | O26:H11 | USA | 2009 |  | 4 mutations | x | 22 mutations | x | x | x | WT | WT | [3] |
| **2602-1** | Dr. Iyoda | ST29C1 | O26:H11 | Japan | 2013 | BD | 4 mutations | x | 22 mutations | x | x | x | WT | WT | [1] |
| **2602-2** | Dr. Iyoda | ST29C1 | O26:H11 | Japan | 2013 | BD | 4 mutations | x | 22 mutations | x | x | x | WT | WT | [1] |
| **2602-3** | Dr. Iyoda | ST29C1 | O26:H11 | Japan | 2013 | BD | 4 mutations | x | 22 mutations | x | x | x | WT | WT | [1] |
| **2602-6** | Dr. Iyoda | ST29C1 | O26:H11 | Japan | 2010 | D | 4 mutations | x | 22 mutations | x | x | x | WT | WT | [1] |
| **2602-8** | Dr. Iyoda | ST29C1 | O26:H11 | Japan | 2013 | BD | 4 mutations | x | 22 mutations | x | x | x | WT | WT | [1] |
| **2602-9** | Dr. Iyoda | ST29C1 | O26:H11 | Japan | 2014 | BD | 4 mutations | x | 22 mutations | x | x | x | WT | WT | [1] |
| **CFSAN025096** | GCA_001571805.1 | ST29C1 | O26:H11 | USA | 2011 |  | 4 mutations | x | 22 mutations | x | x | x | x | x | [6] |
| **CFSAN025102** | GCA_001571915.1 | ST29C1 | O26:H11/H- | USA | 2011 |  | 4 mutations | x | 22 mutations | x | x | x | x | x | [6] |
| **CFSAN025103** | GCA_001571925.1 | ST29C1 | O26:H11/H- | USA | 2011 |  | 4 mutations | x | 22 mutations | x | x | x | x | x | [6] |
| **CFSAN025136** | GCA_001572455.1 | ST29C1 | O26:H11/H- | USA | 2011 |  | 4 mutations | x | 22 mutations | x | x | x | x | x | [6] |
| **34827** | GCA_001039155.1 | ST29C3 | O26:H11 | France | 2012 | HUS | x | x | x | x | x | x | WT | WT | [13] |
| **34870** | GCA_001039145.1 | ST29C3 | O26:H11 | France | 2012 | HUS | x | x | x | x | x | x | WT | WT | [13] |
| **36348** | GCA_001039215.1 | ST29C3 | O26:H11 | France | 2013 | HUS | x | x | x | x | x | x | WT | Stx2d (WT) | [13] |
| **36293** | GCA_001039205.1 | ST29C3 | O26:H11 | France | 2013 | HUS | x | x | x | x | x | x | WT | Stx2d (WT) | [13] |
| **36493** | GCA_001039225.1 | ST29C3 | O26:H11 | France | 2013 | HUS | x | x | x | x | x | x | WT | Stx2d (WT) | [13] |
| **DEC9A** | GCA_000249895.2 | ST29C3 | O26:H11 | USA | 1961 | D | x | x | x | x | x | x | x | x | [8] |
| **DEC9B** | GCA_000249915.2 | ST29C3 | O26:H11 | USA | 1979 | D | x | x | x | x | x | x | x | x | [8] |
| **DEC9C** | GCA_000249935.2 | ST29C3 | O26:H11 | Switzerland | 1952 | D | x | x | x | x | x | x | x | x | [8] |
| **DEC9D** | GCA_000249955.2 | ST29C3 | O26:H11 | Denmark | 1976 | D | x | x | x | x | x | x | x | x | [8] |
| **DEC9E** | GCA_000249975.2 | ST29C3 | O26:H11 | Mexico | 1986 | D | x | x | x | x | x | x | x | x | [8] |

1. Ishijima N, Lee KI, Kuwahara T, Nakayama-Imaohji H, Yoneda S, Iguchi A, Ogura Y, Hayashi T, Ohnishi M, Iyoda S: **Identification of a new virulent clade in enterohemorrhagic *Escherichia coli* O26:H11/H- sequence type 29**. *Sci Rep* 2017, **7**:43136.

2. Ogura Y, Ooka T, Iguchi A, Toh H, Asadulghani M, Oshima K, Kodama T, Abe H, Nakayama K, Kurokawa K *et al*: **Comparative genomics reveal the mechanism of the parallel evolution of O157 and non-O157 enterohemorrhagic *Escherichia coli***. *Proc Natl Acad Sci U S A* 2009, **106**(42):17939-17944.

3. Trees E, Strockbine N, Changayil S, Ranganathan S, Zhao K, Weil R, MacCannell D, Sabol A, Schmidtke A, Martin H *et al*: **Genome sequences of 228 Shiga toxin-producing *Escherichia coli* isolates and 12 isolates representing other diarrheagenic *E. coli* pathotypes**. *Genome Announc* 2014, **2**(4).

4. Mercer RG, Zheng J, Garcia-Hernandez R, Ruan L, Gänzle MG, McMullen LM: **Genetic determinants of heat resistance in *Escherichia coli***. *Front Microbiol* 2015, **6**:932.

5. Hazen TH, Sahl JW, Fraser CM, Donnenberg MS, Scheutz F, Rasko DA: **Refining the pathovar paradigm via phylogenomics of the attaching and effacing *Escherichia coli***. *Proc Natl Acad Sci U S A* 2013, **110**(31):12810-12815.

6. Gonzalez-Escalona N, Toro M, Rump LV, Cao G, Nagaraja TG, Meng J: **Virulence gene profiles and clonal relationships of *Escherichia coli* O26:H11 isolates from feedlot cattle as determined by whole-genome sequencing**. *Appl Environ Microbiol* 2016, **82**(13):3900-3912.

7. Ju W, Cao G, Rump L, Strain E, Luo Y, Timme R, Allard M, Zhao S, Brown E, Meng J: **Phylogenetic analysis of non-O157 Shiga toxin-producing *Escherichia coli* strains by whole-genome sequencing**. *J Clin Microbiol* 2012, **50**(12):4123-4127.

8. Hazen TH, Sahl JW, Redman JC, Morris CR, Daugherty SC, Chibucos MC, Sengamalay NA, Fraser-Liggett CM, Steinsland H, Whittam TS *et al*: **Draft genome sequences of the diarrheagenic *Escherichia coli* collection**. *J Bacteriol* 2012, **194**(11):3026-3027.

9. Grande L, Michelacci V, Bondì R, Gigliucci F, Franz E, Badouei MA, Schlager S, Minelli F, Tozzoli R, Caprioli A *et al*: **Whole-genome characterization and strain comparison of VT2f-producing *Escherichia coli* causing hemolytic uremic syndrome**. *Emerg Infect Dis* 2016, **22**(12):2078-2086.

10. Haugum K, Johansen J, Gabrielsen C, Brandal LT, Bergh K, Ussery DW, Drabløs F, Afset JE: **Comparative genomics to delineate pathogenic potential in non-O157 Shiga toxin-producing *Escherichia coli* (STEC) from patients with and without haemolytic uremic syndrome (HUS) in Norway**. *PLoS One* 2014, **9**(10):e111788.

11. Lambert D, Carrillo CD, Koziol AG, Manninger P, Blais BW: **GeneSippr: a rapid whole-genome approach for the identification and characterization of foodborne pathogens such as priority Shiga toxigenic *Escherichia coli***. *PLoS One* 2015, **10**(4):e0122928.

12. Ferdous M, Zhou K, Mellmann A, Morabito S, Croughs PD, de Boer RF, Kooistra-Smid AM, Rossen JW, Friedrich AW: **Is Shiga toxin-negative *Escherichia coli* O157:H7 enteropathogenic or enterohemorrhagic *Escherichia coli*? Comprehensive molecular analysis using whole-genome sequencing**. *J Clin Microbiol* 2015, **53**(11):3530-3538.

13. Delannoy S, Mariani-Kurkdjian P, Bonacorsi S, Liguori S, Ison SA, Fach P: **Draft genome sequences of human-pathogenic *Escherichia coli* O26:H11 strains carrying the *stx2* gene only and circulating in France**. *Genome Announc* 2015, **3**(4).

14. Delannoy S, Mariani-Kurkdjian P, Webb HE, Bonacorsi S, Fach P: **The mobilome; a major contributor to *Escherichia coli stx2*-positive O26:H11 strains intra-serotype diversity**. *Front Microbiol* 2017, **8**:1625.

15. Gabrielsen C, Drabløs F, Afset JE: **Genome sequences of 11 Shiga toxin-producing *Escherichia coli* strains**. *Genome Announc* 2015, **3**(3).
