# Supplementary figures and images for: Attack of the clones: whole genome-based characterization of two closely related enterohemorrhagic Escherichia coli O26 epidemic lineages
Source: BMC Genomics. 2018 Aug 31;19:647. doi: 10.1186/s12864-018-5045-7 (PMC6119250; doi:10.1186/s12864-018-5045-7)

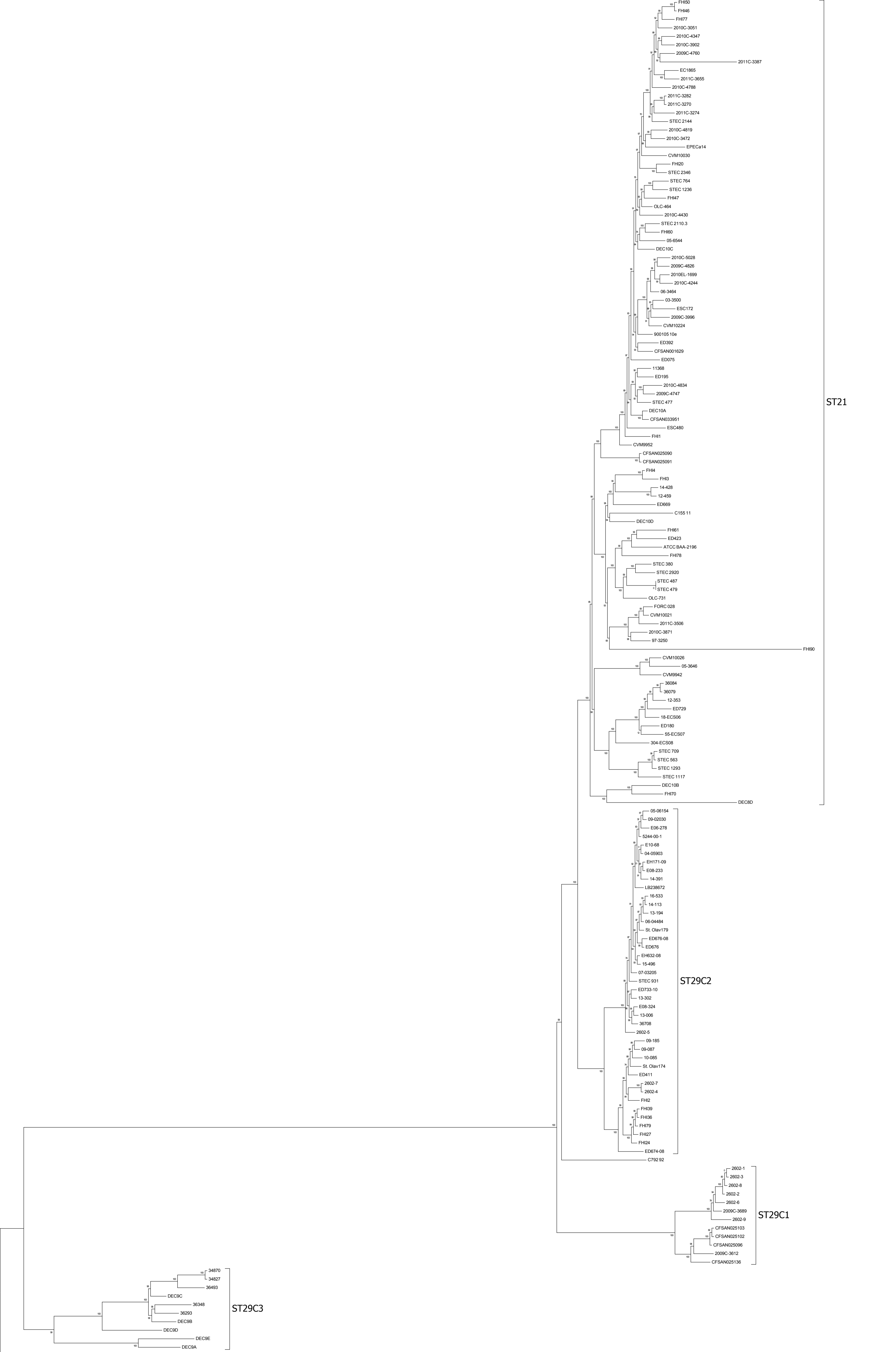

Supplement: Supplementary file 2 — Figure S1. Core genome-derived phylogeny of 159 E. coli O26 isolates. The phylogram (inferred using the CSIPhylogeny pipeline [59]) represents the full version of contracted phylogram presented in Fig. 1. (PDF 32 kb) [file 12864_2018_5045_MOESM2_ESM.pdf]
