# Supplementary material for: Attack of the clones: whole genome-based characterization of two closely related enterohemorrhagic Escherichia coli O26 epidemic lineages
Source: BMC Genomics. 2018 Aug 31;19:647. doi: 10.1186/s12864-018-5045-7 (PMC6119250; doi:10.1186/s12864-018-5045-7)
Supplement: Supplementary file 5 — Figure S2. Alignment of all unique Stx2 protein sequences present in the GenBank. Residue A273 is marked with a red rectangle on the top of alignment. (PDF 6180 kb) [file 12864_2018_5045_MOESM5_ESM.pdf]

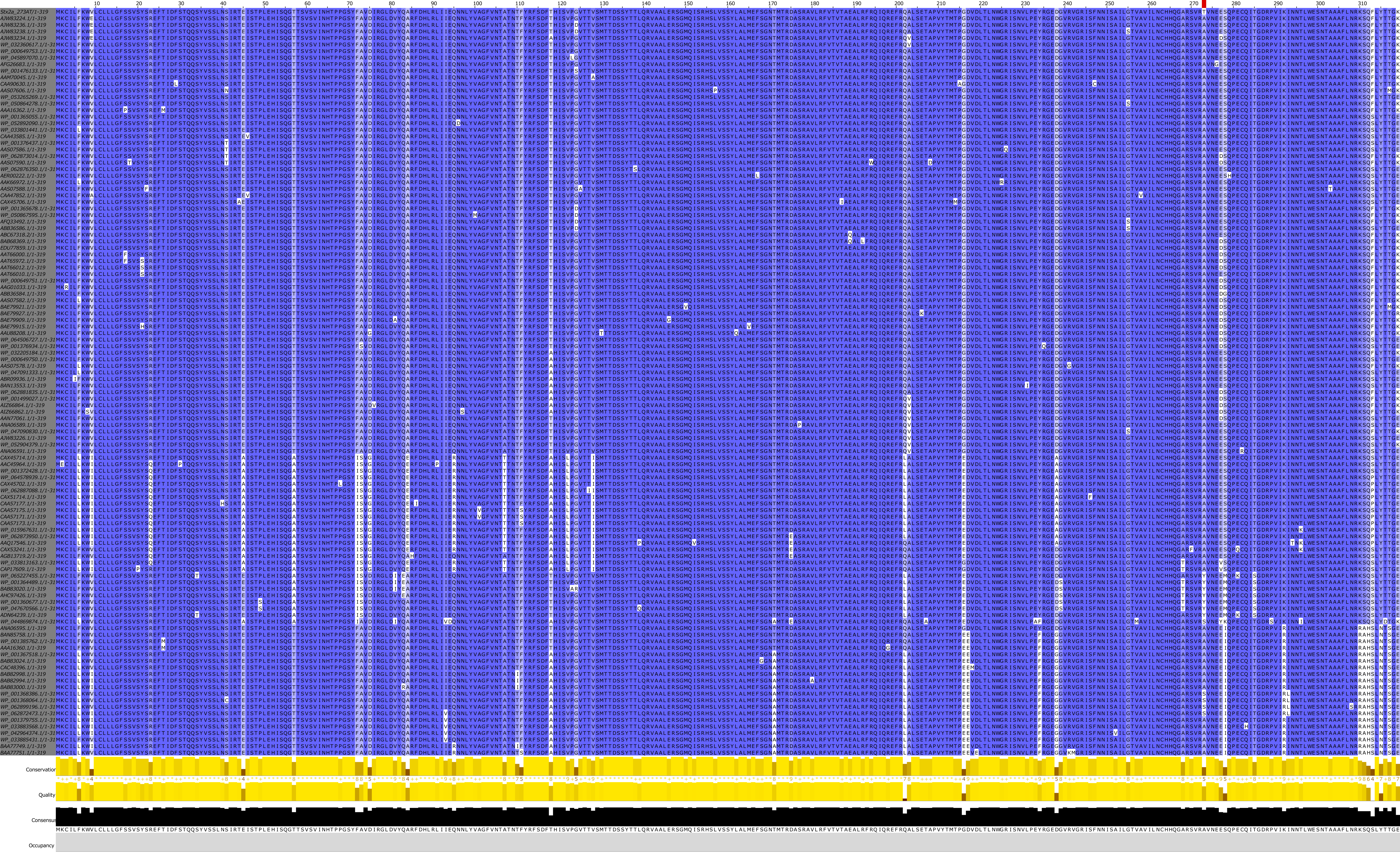
Conservation
Quality
Consensus
Occupancy
